# The Impact of Instant Coffee and Decaffeinated Coffee on the Gut Microbiota and Depression-Like Behaviors of Sleep-Deprived Rats

**DOI:** 10.3389/fmicb.2022.778512

**Published:** 2022-02-25

**Authors:** Xinyi Gu, Shuyi Zhang, Weini Ma, Qixue Wang, Ying Li, Chenyi Xia, Ying Xu, Ting Zhang, Li Yang, Mingmei Zhou

**Affiliations:** ^1^Institute for Interdisciplinary Medicine Sciences, Shanghai University of Traditional Chinese Medicine, Shanghai, China; ^2^Shanghai Frontiers Science Center of TCM Chemical Biology, Institute of Interdisciplinary Integrative Medicine Research, Shanghai University of Traditional Chinese Medicine, Shanghai, China; ^3^School of Rehabilitation Science, Shanghai University of Traditional Chinese Medicine, Shanghai, China; ^4^Department of Physiology, School of Basic Medical Sciences, Shanghai University of Traditional Chinese Medicine, Shanghai, China

**Keywords:** coffee, decaffeinated coffee, sleep deprivation, depression, gut microbiota

## Abstract

**Objective:**

Based on our previous research, chronic paradoxical sleep deprivation (PSD) can cause depression-like behaviors and microbial changes in gut microbiota. Coffee, as the world’s most popular drink for the lack of sleep, is beneficial to health and attention and can eliminate the cognitive sequelae caused by poor sleep. The purpose of this study is to investigate the effects of coffee and decaffeinated coffee on PSD rats.

**Research Design and Methods:**

A total of 32 rats were divided into four groups: control group, PSD model group, conventional coffee group, and decaffeinated coffee group. Behavioral tests, including sucrose preference test, open field test, forced swimming test, and tail suspension test, as well as biochemical detection for inflammatory and antioxidant indexes were performed. The effects of coffee and decaffeinated coffee on the gut microbiota of PSD rats were investigated by 16S rRNA gene sequencing.

**Results:**

Coffee and decaffeinated coffee significantly improved the depression-like behaviors. Moreover, the serum levels of interleukin-6 and tumor necrosis factor alpha were decreased in both coffee and decaffeinated coffee groups, as well as the levels of superoxide dismutase and GSH-Px were increased. Gut microbiota analysis revealed that the abundance of *S24-7*, *Lachnospiraceae*, *Oscillospira*, and *Parabacteroides* were significantly increased in PSD rats, while the abundance of *Akkermansia* and *Klebsiella* were significantly decreased. After the treatment of coffee and decaffeinated coffee, the abundance of the above gut microbiota was all restored in different degrees. Coffee had relatively more significant effects on PSD-induced depressive-like behaviors, while the difference between coffee and decaffeinated coffee was not obvious in correcting the disorder of gut microbiota.

**Conclusions:**

These findings have shown that both coffee and decaffeinated coffee are effective for sleep deprivation-induced depression-like behaviors and the dysbiosis of gut microbiota and indicated that caffeine may be not the only key substance of coffee for regulating gut microbiota.

## Introduction

As the most consumed drink in the world, coffee is second only to water (Butt and Sultan, 2011). In European countries, most adults drink coffee every day (Lopez Garcia et al., 2014). Evidence has been found in recent years that coffee has benefit to the health. People who drink three or four cups of coffee a day have a lower risk of developing type 2 diabetes, which may be due to the presence of green folic acid and caffeine in coffee (George et al., 2008). Moreover, a large amount research reveals that caffeine in the coffee with moderate consumption (three-to-five cups, volume not identified) has the effect of anti-Alzheimer’s disease (Mahshad and Mazen, 2017). Caffeine and coffee with a dose of 600 ml/day are helpful to reduce the risk of depression (Grosso et al., 2016). It is said that caffeine and modafinil could improve neuroinflammation and anxiety during sleep deprivation in rats by inhibiting microglial activation (Meetu et al., 2018), while an excessive dose of caffeine (70 mg/kg) showed anxiogenic effect (Kayir and Uzbay, 2006). Drinking coffee also can enhance cognitive function, in which it is believed that coffee has the benefit for attention and can eliminate cognitive sequelae caused by poor sleep (Franke et al., 2014). However, excessive intake of coffee or caffeine can lead to the development of physiological tolerance, and when a habitual caffeine consumer suddenly reduces or ceases taking caffeine, he or she may experience withdrawal symptoms (Hughes et al., 1998; Stachyshyn et al., 2021).

Coffee contains more than 1,000 different compounds including phenolics, diterpenes, and melanoidins (Renouf et al., 2014), of which about 1% is caffeine (Hoelzl et al., 2010; Kim et al., 2017). Studies have proven that caffeine can improve attention measurement and alertness (Haskell et al., 2005; Childs and Wit, 2006). However, excessive caffeine intake can lead to negative health consequences, such as psychomotor agitation, insomnia, headaches, and gastrointestinal discomfort (Wierzejska, 2012). Caffeine and its metabolites pass freely across the placenta into a fetus. Studies have shown that it may bring damage to the fetus by affecting the expression of genes related to cell damage (Abdelkader et al., 2013). Some recent systematic reviews have shown that moderate intake (three cups a day) of various types of coffee can reduce all-cause mortality in healthy people. These benefits may be due to some biologically active compounds instead of caffeine, mainly phenolic acids and diterpenoids, like cafestol and kahweol (Ding et al., 2015; Tsujimoto et al., 2017; Li et al., 2019). Thus, decaffeinated coffee appeared on the market. Decaffeinated coffee contains only a small amount of caffeine, and the International Coffee Organization defines that the content of caffeine is less than 0.3% in decaffeinated coffee. Chlorogenic acid, a kind of phenolic phytochemicals, can represent the principal non-caffeine components in coffee. Decaffeinated coffee with high chlorogenic acid content improves alertness and reduces negative emotions (Camfield et al., 2013). However, these effects of using chlorogenic acid alone are not obvious, considering that it may be the synergistic effect of non-caffeine compounds in coffee (Camfield et al., 2013). To study the effects of coffee on humans, Bunker and McWilliams proposed a criteria in 1979 to define decaffeinated coffee and coffee, using commercial-branded coffee and espresso (caffeine-containing coffee and caffeine-containing coffee 5 mg per cup) (Adan et al., 2008). Studying the impact of coffee or decaffeinated coffee on cognition can better understand daily habits in life and further clarify the benefits of coffee or decaffeinated coffee (Ho and Chung, 2013).

Sleep deprivation has become a health problem in the modern society (Malinalli et al., 2018). Sleep deprivation can be acute or chronic (Jamie et al., 2019). By definition, 24 h without sleep is acute sleep loss, and less than 6 h of sleep per night for 6 nights or more in a row is considered chronic sleep deprivation (Krishnan et al., 2016). The consequences of sleep deprivation are enormous, especially in mental illness (World Health Organization, 2017). Evidence suggests that rapid eye movement sleep changes occur in most patients with mental illness, such as depression (Benca et al., 1992). Furthermore, sleep deprivation may also occur in the same symptoms (Dieter et al., 2019). A study found that the cytokine secretion induced by low-level exposure of immune cells to bacterial cell wall components contributes to normal sleep patterns, while excessive cytokine levels are associated with disrupted sleep (Galland, 2014). At the same time, recent investigations indicated that the alteration of gut microbiome patterns was evident in people with depression (Barandouzi et al., 2020).

Our previous study found that chronic paradoxical sleep deprivation (PSD) could lead to depression-like behaviors, as well as dysbiosis in the host’s gut microbiota (Ma et al., 2019). PSD could alter monoamine neurotransmitters such as norepinephrine and serotonin, as well as increase neuro-inflammatory cytokines including IL-1β and TNF-α, microglial activation, and neuronal apoptosis in the brain (Daniele et al., 2017; Mengmei et al., 2017). Sleep deprivation also leads to an accumulation of reactive oxygen species and oxidative stress, specifically in the gut (Alexandra et al., 2020). In addition, some preclinical studies have shown that gut microbiota can affect behaviors and brain conditions through neuroimmunity, neuroendocrine, neural, and humoral pathways (Dinan and Cryan, 2013; Kelly et al., 2016). Coffee has been proven to regulate the gut microbiota (González et al., 2020), which depends more on polyphenols and other non-digestible constituents of coffee like polysaccharides and melanoidins (Kolb et al., 2020). Coffee is the most common beverage used to combat fatigue and fatigue caused by sleep deprivation, while decaffeinated coffee is considered as a healthier alternative to traditional coffee. For the purpose of better understanding the effects of these two beverages on sleep deprivation, we first observed the intervention effect of coffee and decaffeinated coffee on the PSD rat model induced by multi-platform technology, mainly related to depression-related behavioral changes and serum inflammation and oxidative stress indicators. Then, we investigated their impacts on the corresponding alterations in the gut microbiota.

## Materials and Methods

### Materials

Commercial instant coffee powder (200 mg/kg; Nestlé, La Tour-de-Peilz, Switzerland) and decaffeinated coffee powder (200 mg/kg; Nestlé, La Tour-de-Peilz, Switzerland) were dissolved in pure water as previously described (Shin et al., 2010; Turner et al., 2012). Each group was administered by gavage in the same volume (0.5 ml/100 g) once a day. Rats in the control and the model group were given saline.

### Animals

A total of 32 inbred-strain male Wistar rats [240 ± 10g, license: SCXK (Hu) 2008-0016] were purchased from Shanghai Sippr-BK Laboratory Animal Co., Ltd. The rats of the same group were housed in animal cages at a density of 4 per cage under standard experimental conditions (room temperature for 24 ± 1°C, relative humidity for 55 ± 15% and 12 h dark/light cycle [07:00–19:00 at 40 w light condition)]. The rats had free access to food and water. Animal welfare and experimental protocols strictly referred to the guide of the care and use of laboratory animals and the ethics and regulations of Shanghai University of Traditional Chinese Medicine. After the rats have been adaptively fed for 1 week, they were randomly divided into four groups as follows: control group (CON), PSD model group (SD), conventional coffee group (CC), and decaffeinated coffee group (DC). Coffee and pure water were administrated on 9:30 a.m. of each day of PSD processing.

### Paradoxical Sleep Deprivation Procedure

The modified multi-platform method was used for PSD processing as described (Cheng et al., 2016). The method was based on the loss of muscle tone that characterizes the rapid eye movement sleep condition or paradoxical sleep. Animals would experience a sudden loss of the sleep cycle when falling into the water, and the method is proved to be feasible in the previous study (Machado et al., 2004). The rats were given free access to water and food in a climate-controlled room (24 ± 1°C, 55 ± 15%) on a 12 h light/dark schedule (light on at 07:00–19:00). The box for PSD was sterilized with 75% alcohol every day. The PSD procedure lasted for 7 days.

### Behavioral Testing

When the PSD was finished on the morning of the eighth day, the behavioral tests of each group were performed under the conditions of dim light and low noise in the following order. Each test started 30 min after the daily administration of coffee.

#### Open Field Test

The open field test (OFT) was performed as previously described (Zhai et al., 2015). The test was performed in a quiet room. The apparatus is a self-made rectangular arena (80 cm × 80 cm × 40 cm), with the floor being divided into 25 equal-size squares, and the side walls were black. After the PSD procedure, the rats were set in the center of the arena one by one to explore for 5 min. The following behaviors were recorded: the number of crossing (grid lines crossed with at least three paws) and the total number of grooming and rearing (defined as standing upright with hind legs). Every grooming or rearing was counted as one point alone, every grid crossed was counted as one point, and the behavioral score was the total number of points. The open-field arena was thoroughly cleaned with 70% ethanol at the interval of each test.

#### Sucrose Preference Test

The sucrose preference test (SPT) was performed as previously described (Zhai et al., 2015). All rats were reared in a single cage during the experiment. The rats were trained to adapt by exposing them to two bottles (one containing 1% sucrose solution and the other containing tap water) for 24 h. Then, the test was performed after 4 h of water deprivation. Two bottles (one with 1% sucrose solution and the other with tap water) were weighed and presented to each rat for 1 h. The position of the two bottles was randomly determined. Sucrose solution and tap water consumption (g) were measured, and the sucrose preference was calculated using the equation: sucrose solution (g)/[sucrose solution (g) + water (g)] × 100%.

#### Forced Swimming Test

After administration, each rat was placed in a transparent container (50 cm in height, 18 cm in diameter) with water in 30 cm depth (25 ± 1°C). The test lasted 6 min, and the immobility time during the final 4 min was recorded by the person blinded to the purpose of the experiment. The immobility state was defined as the state of rats floating in the water and only keeping the head above the water without struggling or any motions. The test time was from 14:00 to 18:00, and the water was changed after each test.

#### Tail Suspension Test

Each rat was individually suspended 50 cm above the floor by the tail, using a tape. The test lasted 6 min, and the immobility time during the final 4 min was recorded by the person blinded to the purpose of the experiment. The immobility state was defined as the state only when rats remain completely motionless. The test time was from 14:00 to 18:00.

### Sample Collection

After the last sleep deprivation and administration were performed, the rats were individually housed in a metabolic cage, which could separate and collect urine and feces. Fresh feces were collected continuously with sterile operation (Eriksson et al., 2004; Ward and Hubscher, 2012), and stored at −80°C for subsequent analysis. Then rats were sacrificed, and blood was collected from the abdominal aorta. Blood was centrifuged at 4°C at 3,000 r/min for 15 min (Centrifuge 5702R, Eppendorf), and the supernatants were stored at −80°C prior to use.

### Biochemical Parameters in Serum

Serum samples were thawed on ice. The levels of interleukin-6 (IL-6), tumor necrosis factor alpha (TNF-α), superoxide dismutase (SOD), and glutathione peroxidase (GSH-Px) in serum samples were detected by commercial enzyme linked immunosorbent assay (ELISA) kits, referring to the instructions provided by the manufacturer (Nanjing Jiancheng Bioengineering Institute, Nanjing, China).

### Microbial Community Profiling

The E.Z.N.A. ^®^ soil DNA Kit (Omega Bio-Tek, Norcross, GA, United States) was used to extract the microbial community genomic DNA from fecal samples. The microbial DNA regions V3–V4 of the bacterial 16S rRNA gene were amplified with primer pairs 338F (5′-ACTCCTACGGGAGGCAGCAG-3′) and 806R (5′-TACHVGGGTWTCTAAT-3′) (Sinha et al., 2017). The PCR product was extracted from 2% agarose gel and purified using the AxyPrep DNA Gel Extraction Kit (Axygen Biosciences, Union City, CA, United States). Purified amplicons were pooled in equimolar and paired-end sequences on an Illumina MiSeq PE300 platform/NovaSeq PE250 platform (Illumina, San Diego, CA, United States) according to the standard protocols by Majorbio Bio-Pharm Technology Co., Ltd. (Shanghai, China). The raw reads were deposited into the NCBI Sequence Read Archive database (Accession Number: SRP337042; PRJNA762663).

Fastp software (^1^ version 0.20.0) and FLASH software (^2^ version 1.2.7) were used for the quality control of the original sequencing sequence and splicing (Magoč and Salzberg, 2011; Shifu et al., 2018). UPARSE software (^3^ version 7.1) was used to perform an operational taxonomic unit (OTU) clustering of sequences based on 97% similarity, as well as to eliminate chimeras (Stackebrandt and Goebel, 1994; Edgar, 2013). The Venny 2.1^4^ was employed to map the Venn diagram of OTUs among four groups. Alpha diversity index (Chao1 index, Ace index, Simpson index, and Shannon index) was based on Mothur 9 (version 1.30.2^5^), and beta diversity was performed based on partial least squares discriminant analysis (PLS-DA) for assessing the clustering patterns on the weighted UniFrac matrices and the ANOSIM function of QIIME 11 (version 1.9.1^6^).

### Data Statistical Analysis

All the data were presented as mean ± standard deviation. The statistical analyses were carried out using IBM statistical product and service solutions (SPSS) Statistic Version 21.0 (SPSS; IBM, Armonk, NY, United States). One-way ANOVA test and two tailed Student’s *t*-test were used to analyze significant differences between the two groups. A *p*-value of less than 0.05 or 0.01 was considered statistical significance.

## Results

### Coffee and Decaffeinated Coffee Improved the Depressive-Like Behaviors in PSD Rats

**FIGURE 1:**
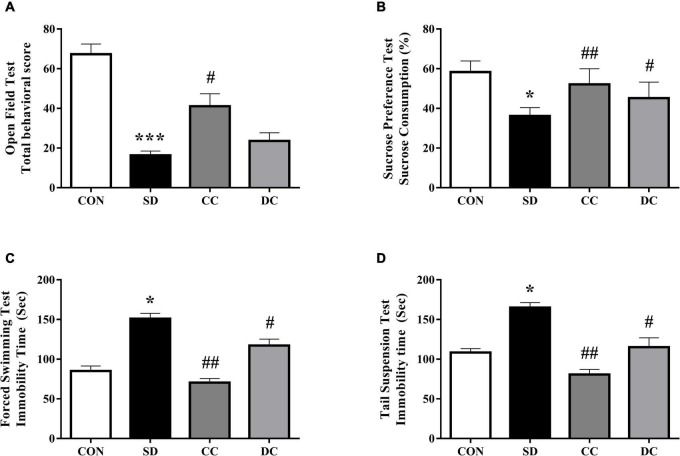
Effects of coffee and decaffeinated coffee on different behaviors in the PSD model. Total behavior score **(A)**, sucrose consumption **(B)**, immobility time of FST **(C)**, immobility time of TST **(D)**. Values were expressed as means ± SEM (*n* = 8). *P < 0.05, ***P < 0.001 significant differences compared to the control group; ^#^*P* < 0.05, ^##^*P* < 0.01 significant differences compared to the SD group. CON, control group; SD, PSD model group; CC, conventional coffee group; DC, decaffeinated coffee group.

After 7 days of sleep deprivation procedure, as showed in Figure 1A, the total behavioral score in the PSD model group was significantly lower than the control group (*P* < 0.001). Conventional coffee significantly increased the total behavioral score (*P* < 0.05), while decaffeinated coffee also increased the score (*P* > 0.05).

The results of SPT showed the effects of coffee and decaffeinated coffee on the changes of sucrose consumption (Figure 1B). Compared to the control group, sucrose consumption was significantly reduced in the PSD model group (*P* < 0.05). However, the sucrose consumption was significantly higher after the treatment of conventional coffee and decaffeinated coffee than in the model group (*P* < 0.01, *P* < 0.05).

Chronic PSD remarkably increased immobility time in forced swimming test (FST) and tail suspension test (TST) (Figures 1C,D) (*P* < 0.05). The immobility time in the conventional coffee and decaffeinated coffee group was significantly lower than the PSD model group (*P* < 0.01, *P* < 0.05).

### Coffee and Decaffeinated Coffee Ameliorate the Level of Inflammatory and Antioxidant Factors in PSD Rats

The effects of conventional coffee and decaffeinated coffee on inflammatory factors and antioxidant factors in the serum of PSD rats were investigated (Table 1). Compared with the control group, the levels of IL-6 and TNF-α in PSD rats were significantly increased (*P* < 0.001, *P* < 0.01). IL-6 and TNF-α levels were remarkably decreased after the treatment of conventional coffee (*P* < 0.01, *P* < 0.05), and the level of TNF-α was significantly decreased with the treatment of decaffeinated coffee (*P* < 0.05). Furthermore, the levels of SOD and GSH-Px in the model group were much lower than in the control group (*P* < 0.01, *P* < 0.001). The levels of SOD and GSH-Px were significantly increased with the treatment of conventional coffee (*P* < 0.05, *P* < 0.01), and the level of GSH-Px was significantly increased after the treatment of decaffeinated coffee (*P* < 0.05).

**TABLE 1:** Inflammatory and antioxidant factors in the serum.

Parameter	n	CON	SD	CC	DC
IL-6 (pg/ml)	8	4.19 ± 1.04	17.58 ± 3.71***	10.25 ± 3.01^##^	14.03 ± 2.42
TNF-α (pg/ml)	8	5.07 ± 0.79	12.57 ± 2.67**	8.34 ± 3.16^#^	9.22 ± 2.66^#^
SOD (U/ml)	8	156.32 ± 11.61	104.57 ± 13.45**	140.35 ± 15.26^#^	137.88 ± 16.39
GSH-Px (U/ml)	8	319.23 ± 22.70	167.53 ± 17.13***	217.53 ± 27.35^##^	193.50 ± 23.62^#^

*Values were expressed as means ± SEM.*

**P < 0.01, ***P < 0.001 significant differences compared to the control group; ^#^P < 0.05, ^##^P < 0.01 significant differences compared to the SD group.

*CON, control group; SD, PSD model group; CC, conventional coffee group; DC, decaffeinated coffee group.*

### Effects of Coffee and Decaffeinated Coffee on the Gut Microbiota Composition

#### OTU Classification Statistics

The number of OTUs of each sample, as well as common and unique OTUs, was shown by the Venn diagram (Supplementary Figure 1), which could describe sample similarity and overlap intuitively (Cheng et al., 2016). The OTUs alone in each group were as follows: CON group 479; SD group 663; CC group 494; and DC group 465. The total number of OTU in each group was 3,359 (CON), 3.814 (SD), 3,458 (CC), and 3,430 (DC). Therefore, the ratio of the OTU alone in each group to the total OTU was 3.41, 4.72, 3.51, and 3.31%, respectively. A total of 1,343 OTUs were shared among the four groups. The results showed that the out number of the PSD model group increased, while both coffee and decaffeinated coffee treatments could reduce it.

#### The Diversity of the Gut Microbiota

**FIGURE 2:**
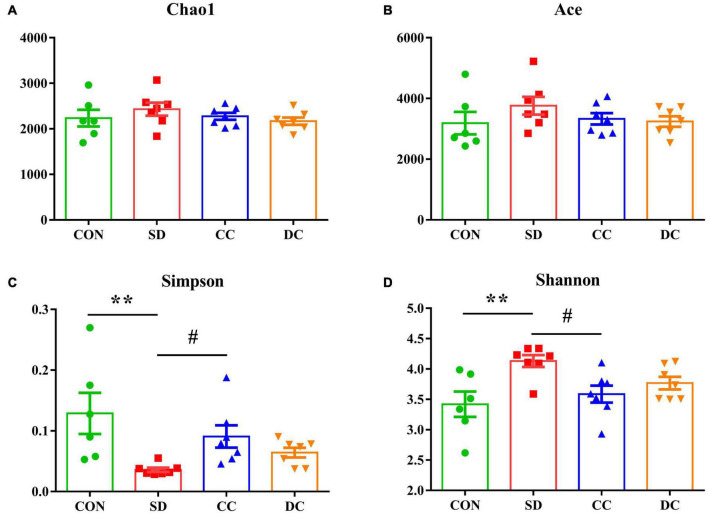
Alpha diversity in each group. Chao1 diversity **(A)**; Ace diversity **(B)**; Simpson diversity **(C)**; Shannon diversity **(D)**. Values were expressed as means ± SEM (*n* = 6 or 7). **P < 0.01 significant differences between the SD group and the control group; ^#^*P* < 0.05 significant differences of the CC group or DC group as compared to the SD group. CON, control group; SD, PSD model group; CC, conventional coffee group; DC, decaffeinated coffee group.

The Chao1 index, Ace index, Simpson index, and Shannon index were selected to analyze the alpha diversity (Figure 2). The value of Simpson was significantly decreased, and the value of Shannon was significantly increased in the PSD model group compared with the control group (*P* < 0.01, *P* < 0.01). After the administration of conventional coffee, the value of Simpson was significantly increased and the value of Shannon was significantly lower than that in the PSD model group (*P* < 0.05, *P* < 0.05), while those values had no significantly changes with the administration of decaffeinated coffee.

**FIGURE 3:**
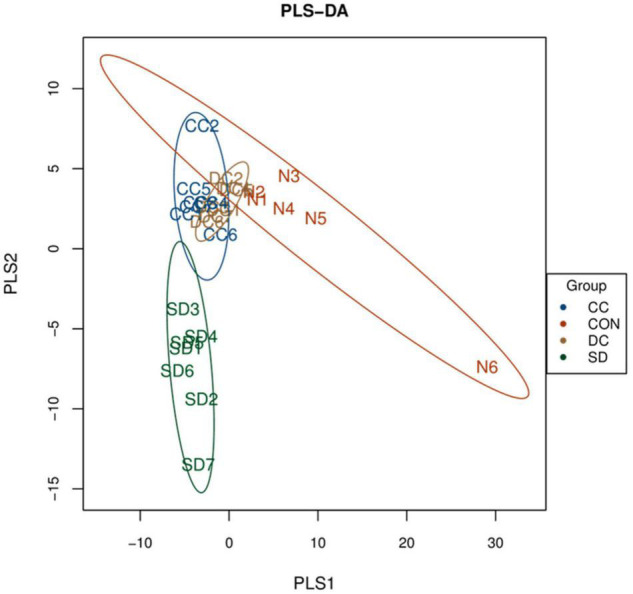
PLS-DA score plots for beta diversity of the microbial community among each group (*n* = 6 or 7). CON, control group; SD, PSD model group; CC, conventional coffee group; DC, decaffeinated coffee group.

PLS-DA analysis was performed to analyze the beta diversity among the four groups. As shown in Figure 3, the composition of the gut microbiota of the control group and the PSD model group was significantly separated and changed. In addition, the composition of the gut microbiota of the conventional coffee group and the decaffeinated coffee group was similar, and both tended to be closer to the control group than the model group. These results indicated that PSD caused disturbances in the gut microbiota. The treatment of conventional coffee and decaffeinated coffee could improve the PSD-induced changes in the gut microbiota, and both had similar therapeutic effects.

#### Analysis of the Gut Microbiota Composition

**FIGURE 4:**
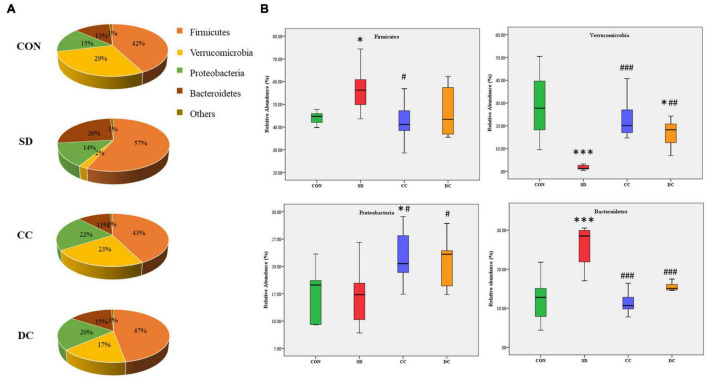
Relative abundances profiling at the phylum level of gut microbiota. **(A)** Pie chart of the relative abundances of gut microbiota in each group; **(B)** the relative abundances of *Firmicutes*, *Verrucomicrobia*, *Proteobacteria*, and *Bacteroidetes*. Values were expressed as means ± SEM (*n* = 8). *P < 0.05, ^***^P < 0.001 significant differences compared to the control group; ^#^*P* < 0.05, ^##^*P* < 0.01, ^###^*P* < 0.001 significant differences compared to the SD group. CON, control group; SD, PSD model group; CC, conventional coffee group; DC, decaffeinated coffee group.

At the phylum level, gut microbiota was mainly composed of *Firmicutes*, *Verrucomicrobia*, *Proteobacteria*, and *Bacteroidetes* (Figure 4A). Compared with the control group, the abundance of *Firmicutes* and *Bacteroidetes* was remarkably elevated in the model group (*P* < 0.05, *P* < 0.001). After the treatment of conventional coffee, the abundance of *Firmicutes* and *Bacteroidetes* was significantly reduced (*P* < 0.05, *P* < 0.001), and after the treatment of decaffeinated coffee, only the abundance of *Bacteroidetes* had a significant decrease (*P* < 0.001). Moreover, compared with the control group, the abundance of *Verrucomicrobia* was remarkably decreased in the model group (*P* < 0.001). With the treatment of conventional coffee and decaffeinated coffee, the abundance of *Verrucomicrobia* was significantly reversed (*P* < 0.001, *P* < 0.01). After decaffeinated coffee treatment, the abundance was still much lower than the control group (*P* < 0.05). For the abundance of *Proteobacteria*, there was no significant difference between the model group and the control group, and after the administration of conventional coffee and decaffeinated coffee, the abundance was much higher than the model group (*P* < 0.05, *P* < 0.05) and the control group (*P* < 0.05, no statistical significance) (Figure 4B).

**FIGURE 5:**
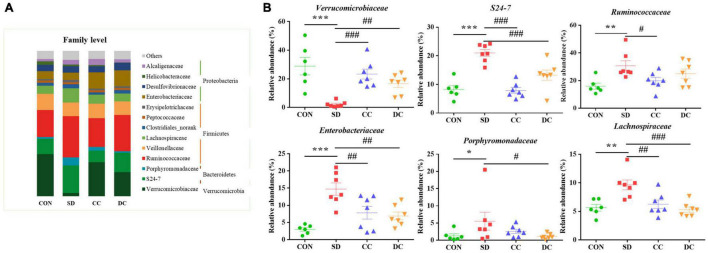
Bacterial taxonomic profiling at the family level of gut microbiota. **(A)** The relative abundance of *Verrucomicrobiaceae*, *S24-7*, *Ruminococcaceaea*, *Enterobacteriaceae*, *Porphyromonadaceae*, and *Lachnospiraceae*; **(B)** values were expressed as means ± SEM (*n* = 8). *P < 0.05, **P < 0.01, ***P < 0.001 significant differences compared to the control group; ^#^*P* < 0.05, ^##^*P* < 0.01, ^###^*P* < 0.001 significant differences compared to the SD group. CON: control group; SD: PSD model group; CC: conventional coffee group; DC: decaffeinated coffee group.

At the family level, the abundance of *Verrucomicrobiaceae* (*P* < 0.001), *S24-7* (*P* < 0.001), *Ruminococcaceaea* (*P* < 0.01), *Enterobacteriaceae* (*P* < 0.001), *Porphyromonadaceae* (*P* < 0.05), and *Lachnospiraceae* (*P* < 0.01) showed a significant difference between the PSD and normal group (Figures 5A,B). Compared with the PSD model group, those relatively abundance except *Porphyromonadaceae* were significantly reversed after the treatment of conventional coffee, and those relatively abundance except *Ruminococcaceae* were significantly reversed after the treatment of decaffeinated coffee (Figure 5B).

**FIGURE 6:**
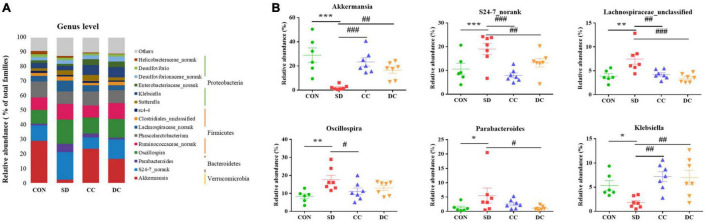
Bacterial taxonomic profiling at the genus level of gut microbiota. **(A)** The relative abundance of *Akkermansia*, *S24-7_norank*, *Lachnospiraceae_unclassified*, *Oscillospira*, *Parabacteroides*, and *Klebsiella*; **(B)** values were expressed as means ± SEM. (*n* = 8). *P < 0.05, **P < 0.01, ***P < 0.001 significant differences compared to the control group; ^#^*P* < 0.05, ^##^*P* < 0.01, ^###^*P* < 0.001 significant differences compared to the SD group. CON, control group; SD, PSD model group; CC, conventional coffee group; DC, decaffeinated coffee group.

At the genus level, *Akkermansia* (*P* < 0.001), *S24-7_norank* (*P* < 0.001), *Lachnospiraceae_unclassified* (*P* < 0.01), *Oscillospira* (*P* < 0.01), *Parabacteroides* (*P* < 0.05), and *Klebsiella* (*P* < 0.05) were the differential microbiota of the top six highest contents, between the PSD and normal group (Figures 6A,B). After the treatment of conventional coffee, the abundance of *Akkermansia* (*P* < 0.001), *S24-7_norank* (*P* < 0.001), *Lachnospiraceae_unclassified* (*P* < 0.01), *Oscillospira* (*P* < 0.05), and *Klebsiella* (*P* < 0.01) significantly reversed, while the abundance of *Akkermansia* (*P* < 0.01), *S24-7_norank* (*P* < 0.01), *Lachnospiraceae_unclassified* (*P* < 0.001), *Parabacteroides* (*P* < 0.05), and *Klebsiella* (*P* < 0.01) significantly reversed after the treatment of decaffeinated coffee (Figure 6B).

## Discussion

Chronic sleep deprivation is a stressor that impairs the brain function and causes cognitive impairment, as well as increases oxidative stress and the risk for Alzheimer’s disease or depression (McEwen, 2006; Ma et al., 2019). The results of the behavior tests in our study showed the similar depression-like behaviors after PSD induced, and the symptom could be improved in a different extent with the treatment of conventional coffee and decaffeinated coffee (Figure 1). It has been reported that caffeinated coffee and caffeine were beneficial to depression-like behaviors, while the effect of decaffeinated coffee was not obvious (Paz-Graniel et al., 2020; Xue et al., 2020).

Elevated levels of IL-6 and TNF-α, as well as a negative feedback of SOD and GSH-Px levels in the PSD group (Table 1) confirmed that sleep deprivation induced inflammation and oxidative stress. Both IL-6 and TNF-α are the multifunctional cytokines involved in the regulation of the immune response and inflammation, and the production of these pro-inflammatory cytokines could be increased by different stimuli (Akira et al., 1990; Schindler et al., 1990). As one of the stimuli, sleep disturbance was associated with disorders related to inflammation, such as cardiovascular disease, arthritis, diabetes mellitus, and certain cancers (Michael et al., 2015). In addition, evidence suggested that sleep deprivation would promote oxidative stress (Villafuerte et al., 2016). In our study, the levels of IL-6, TNF-α, SOD, and GSH-Px were all reversed after the treatment of conventional coffee and decaffeinated coffee, while the effect of coffee was relatively more significant than that of decaffeinated coffee (Table 1). The anti-inflammatory effect of coffee has been widely reported (Yamashita et al., 2012; Erikka et al., 2015). In our study, both in the caffeinated coffee group and the decaffeinated coffee group, a decrease in the levels of pro-inflammatory factors was observed, and the therapeutic effect of caffeinated coffee was more obvious, which indicated that not only caffeine but also the other ingredients in coffee played a role in promoting anti-inflammatory effect, such as chlorogenic acid (Dong et al., 2019). Chlorogenic acid can inhibit protein tyrosine phosphatase 1B to reduce the expression of pro-inflammatory cytokine genes (Giuseppe et al., 2017). Research on the effect of coffee and its biologically active substances such as caffeine, phenolic compounds, diterpenoids. and soluble fiber on oxidative stress is increasing these years (Martini et al., 2016). It has been reported that chronic coffee and caffeine ingestion would increase the activity of SOD, as well as protect the antioxidant system in the brain (Abreu et al., 2011). Light-dark roasted coffee (rich in chlorogenic acid) showed a more significant effect in increasing the level of SOD (Kotyczka et al., 2011). Evidence showed that some diterpenoids (kafestol and kahweol) in coffee could increase the concentration of GSH (Scharf et al., 2001). Furthermore, caffeine also has the exact effect of antioxidant and anti-inflammatory (Daniela et al., 2017; Soohan et al., 2017; Haroon et al., 2019).

Coffee has been confirmed to confer various health benefits, and drinking coffee can alter a host’s gut microbiota (Chong et al., 2020). Coffee may partly depend on the modulation of gut microbiota to influence health (González et al., 2020). It has been proven that polyphenols in coffee can change the environment, or they can be catabolized by gut microbiota (Aura, 2008; Couteau et al., 2010). In the microbial community profiling, both caffeinated coffee and decaffeinated coffee could reduce the number of OTUs that increased after PSD induction. Only the Simpson index and Shannon index in alpha diversity index were restored significantly after coffee treatment. In the beta diversity analysis, there was a significant separation between the control group and the model group. With the treatment of coffee and decaffeinated coffee, the composition of the gut microbiota tended to approach the control group, which indicated that both coffee and decaffeinated coffee restored the composition of the gut microbiota of PSD rats. In our results, the levels of *Akkermansia* (Phylum *Verrucomicrobia*) and *Klebsiella* (Phylum *Proteobacteria*) were significantly decreased in the rats with sleep deprivation (Figure 6B). As a Gram-negative pathogen, *Klebsiella* could cause a variety of infectious diseases (Guoying et al., 2020) and has the effect of inducing the inflammatory response (Atarashi et al., 2017; Marjolaine et al., 2019). It has been reported that when the *Proteobacteria* increases, intestinal mucus would decrease, resulting in damage to the intestinal barrier and low-grade inflammation (Shin et al., 2015). In our results, sleep deprivation caused an increase in the expression of inflammatory factors, while a decrease in the pro-inflammatory bacteria *Proteobacteria*. This indicated that the inflammation caused by sleep deprivation might be not through the gut microbiota pathway. The decrease of *Akkermansia* is commonly observed in the symptom of sleep deprivation (Heintz-Buschart et al., 2018; Ting et al., 2019). *Akkermansia* is known as a beneficial microbe that is inversely associated with obesity, diabetes, cardiometabolic diseases, and low-grade inflammation (Plovier et al., 2016; Patrice and Willem, 2017). With treatment of conventional coffee and decaffeinated coffee, both levels of *Akkermansia* were increased significantly, and the effect of coffee was more obvious (Figure 6B). A study has found that coffee has antibacterial effects and prebiotic function (Nakayama and Oishi, 2013). Compared to caffeine, which is rapidly absorbed and degraded in the upper digestive tract, polyphenols are difficult to be absorbed in the gastrointestinal tract. This caused most of the polyphenols to enter the intestine and affect the gut microbiota and mucosal cells and increase of the abundance of *Akkermansia* in the intestine (Yuji et al., 2018). A study has shown that tea is rich in polyphenols and caffeine plays a key role in remodeling the disturbed gut microbiota (including *Akkermansia*), which may be a synergistic effect (Xiaoyu et al., 2018).

Our results illustrated that the abundance of *Parabacteroides* (Phylum *Bacteroidetes*) in the PSD group was significantly higher than the normal control. As a kind of Gram-negative bacteria, *Parabacteroides* contains lipopolysaccharide, which is a typical potent endotoxin that can induce strong pro-inflammatory reactions in the host (Kaisa et al., 2020). *Akkermansia* and *Klebsiella*, also Gram-negative bacteria, had lower levels in the PSD group, compared to the normal control, and high levels of inflammatory factors were detected after PSD intervention. These indicated that the inflammatory response might not necessarily be caused by *Parabacteroides*, *Akkermansia*, and *Klebsiella*, but rather be caused by the release of lipopolysaccharide from other types of Gram-negative bacteria or other stress response in the host. In addition, a high level of *Parabacteroides* can be found in patients with depression (Barandouzi et al., 2020), and our results also showed depression-like behaviors after PSD induced. With the treatment of decaffeinated coffee, the decrease of the level of *Parabacteroides* was relatively more significant than the conventional coffee group. Polyphenols in the decaffeinated green tea and black tea could regulate the abundance of *Parabacteroides* and induced weight loss (Rothenberg et al., 2018). For example, chlorogenic acid can protect intestinal integrity and reduce inflammation by inhibiting the growth of *Bacteroides* and the accumulation of *Bacteroides*-derived lipopolysaccharide (Yan et al., 2020). In addition, evidence has indicated that both caffeinated and decaffeinated coffee have a similar anti-inflammatory effect (Dong et al., 2019), and not only caffeine, but also some phenols including pyrocatechol, chlorogenic acid, and so on, can prevent the inflammatory responses (Hwang et al., 2016; Stefanello et al., 2018; Megumi et al., 2020). Therefore, caffeine and phenols exert anti-inflammatory effects by affecting different gut microbiota.

In our study, the relative abundance of *Lachnospiraceae* (Phylum *Firmicutes*), *S24-7* (Phylum *Bacteroidetes*), and *Oscillospira* (Phylum *Firmicutes*) were significantly increased in the SD group (Figure 6B). *Lachnospiraceae* and *S24-7* impact their hosts by producing short-chain fatty acids (SCFAs) (Jennifer et al., 2017; Matthew et al., 2020). In patients with ulcerative coliti, an increase in the level of *Lachnospiraceae* could be observed (Schirmer et al., 2019), which could be explained by the fact that stress increases the abundance of *Lachnospiraceae* (Li et al., 2017). As shown in our study, after the stress of sleep deprivation, the level of *Lachnospiraceae* increased significantly. However, as a butyric acid-producing bacteria, increasing the content of *Lachnospiraceae* also has a beneficial effect to the body (Huws et al., 2011; Dong et al., 2020). *Oscillospira* can be seen in the obese animals, which ferments polysaccharide into SCFAs (Na et al., 2018). In addition, *Oscillospira* is correlated with inflammatory disease (Uri et al., 2017). SCFAs are involved in the regulation of the gut immune system, and the production of the SCFAs is one of the crucial ways that gut microbiota affect the hosts (Jost et al., 2020). Besides, SCFAs play an important role in host defense and immunity, including anti-inflammation and anti-oxidant activities. The increase of these gut microbiota related to fatty acid metabolism indicated that sleep deprivation would lead to metabolism disorders, inflammation, and oxidative stress. After the administration of conventional coffee and decaffeinated coffee, the relative abundance of these gut bacteria decreased in different degrees, and coffee had a relatively more significant effect. Caffeine consumption could attenuate the increase in *Firmicutes*-to-*Bacteroidetes* ratio in high-fat-fed rats (Cowan et al., 2014). Interestingly, some recent studies have now shown that the increase in this ratio has no relation with the presence of high- fat diet but only related to the content of dietary fiber (Dalby et al., 2017; Singh et al., 2020). Moreover, due to lifestyle-associated factors such as diet, physical activity, food additives, and contaminants, the relative abundance of the *Firmicutes* and *Bacteroidetes* varies greatly among individuals (Magne et al., 2020). Caffeine intake is linked to weight loss and the regulation of lipid metabolism, partly through its inhibition of adipogenesis-related factors (Su et al., 2013). It has been reported that some phenols and caffeine were directly associated with *Bacteroides* group levels (González et al., 2020). A study has found that caffeine and chlorogenic acid in coffee have a partially positive effect to the SCFAs in plasma (Kazuchika et al., 2018). The reaction of polyphenols in tea with residual carbohydrates and gut microbiota within the colon produce SCFAs, which enhance lipid metabolism (Rothenberg et al., 2018).

As shown in the previous studies, the differential gut microbiota in our study is commonly seen in patients with depression (Zheng et al., 2016; Cheung et al., 2019). Gut microbiota influences the emotional behavior by affecting the interactions of the gut–brain axis (Emeran et al., 2015), and sleep deprivation would increase the risk of depression (Robert and Hao, 2014). Chronic PSD could bring about depression-like performance, which is proven by our study and previous researches (Ma et al., 2019; Rahmani et al., 2020). In the behavior tests of our study, the effect of coffee to improve depression is more significant than the decaffeinated coffee. The main difference between the two drinks is the caffeine content. Caffeine is an alkaloid and modulates the dopaminergic activity through nonspecific antagonism against A1/A2 adenosine receptors (Navarro et al., 2018). A moderate intake of caffeine has a stimulating effect on the central nervous system and can improve psychomotor activities (Adan et al., 2008). Furthermore, coffee contains many functional components other than caffeine, such as chlorogenic acid, ferulic acid, nicotinic acid, trigonelline, quinolinic acid, tannic acid, and pyrogallic acid. These compounds have anti-inflammatory or antioxidative effects. Chlorogenic acid and trigonelline, which are present in high amounts in coffee, have an anti-inflammatory effect (Godos et al., 2014), and it has been found that low-grade inflammation seems to be related to the pathogenesis of depression (Sanchez Villegas and Martínez González, 2013). From our results of the treatment with decaffeinated coffee, we know that polyphenols in coffee also can improve depression-like behaviors by affecting the gut microbiota associated with inflammation such as *Akkermansia*, *Klebsiella*, and *Parabacteroides*. On the other hand, it has been suggested that patients with depression have increased oxidative stress and decreased antioxidant defense (Black et al., 2015) and antioxidant is an important aspect of treating depression (Vaváková et al., 2015). Coffee is rich in polyphenols, which not only has an anti-inflammatory effect but also has antioxidant activity (Godos et al., 2014; Martini et al., 2016). In our study, the abundance of the gut microbiota related to oxidative stress such as *S24-7*, *Lachnospiraceae*, and *Oscillospira*, could be revised by both coffee and decaffeinated coffee. Thus, it is demonstrated that the anti-inflammatory and antioxidant effects of polyphenols may be through regulating the gut microbiota, further playing a role in the treatment of depression induced by chronic PSD.

Some limitations must be considered when interpreting our results. The manufacturing procedure of the instant coffee powder we used in the experiment involves the aqueous extraction of soluble coffee components followed by drying to form a soluble powder. Although there are no other food additives, the loss of volatile aroma compounds during concentration through evaporation will lower product quality (Beverly et al., 2020), and at the same time, the results of the study could not fully represent the activity of coffee. In addition, the study lacks the analysis of the components of the samples, although there are other relevant literatures for reference, as well as the analysis of the metabolites of gut microbiota (such as SCFAs), these need to be strengthened in future research to better judge the role of gut microbiota in coffee on sleep deprivation.

## Conclusion

In this study, 16S rRNA gene sequencing was applied to assess the effects of conventional coffee and decaffeinated coffee on the gut microbial community profiling. Our results revealed that the administration of conventional coffee and decaffeinated coffee ameliorated depression-like behaviors in rats of PSD induced, as well as the changed levels of IL-6, TNF-α, SOD, and GSH-Px. The effect of conventional coffee was relatively obvious than that of decaffeinated coffee. In microbiome analysis, PSD disturbed the composition of gut microbiota, including *Akkermansia*, *S24-7*, *Lachnospiraceae*, *Oscillospira*, *Parabacteroides*, and *Klebsiella*. Both the treatment of conventional coffee and decaffeinated coffee could restore the abundance levels of these gut microbiota. In a word, both coffee and decaffeinated coffee are effective for sleep deprivation-induced depression-like behaviors and the dysbiosis of gut microbiota. It implies that caffeine is not the only key substance of coffee in the regulation of PSD induced gut microbiota disorder.

## Data Availability Statement

The datasets presented in this study can be found in online repositories. The names of the repository/repositories and accession number(s) can be found below: https://www.ncbi.nlm.nih.gov/, PRJNA762663.

## Ethics Statement

The animal study was reviewed and approved by Animal Experiment Center, Shanghai University of Traditional Chinese Medicine.

## Author Contributions

YX, TZ, MZ, CX, and LY accomplished the conception and design of the research. XG, SZ, WM, QW, and YL performed the experiments. XG and SZ prepared the figures. XG, WM, and SZ analyzed and interpreted the data. XG, WM, and YL drafted the manuscript. MZ edited and revised the manuscript. MZ and LY approved final version of the manuscript. All authors read and approved the final manuscript.

## Conflict of Interest

The authors declare that the research was conducted in the absence of any commercial or financial relationships that could be construed as a potential conflict of interest.

## Publisher’s Note

All claims expressed in this article are solely those of the authors and do not necessarily represent those of their affiliated organizations, or those of the publisher, the editors and the reviewers. Any product that may be evaluated in this article, or claim that may be made by its manufacturer, is not guaranteed or endorsed by the publisher.

## Footnotes

1
https://github.com/OpenGene/fastp

2
http://www.cbcb.umd.edu/software/flash

3
http://drive5.com/uparse/

4
http://bioinfogp.cnb.csic.es/tools/venny/index.html

5
https://www.mothur.org/wiki/Download_mothur

6
http://qiime.org/install/index.html

## Abbreviations

FST: forced swimming test
GSH-Px: glutathione peroxidase
IL-6: interleukin-6
OFT: open field test
PSD: paradoxical sleep deprivation
SOD: superoxide dismutase
SPT: sucrose preference test
TNF-α: tumor necrosis factor alfa
TST: tail suspension test.
