# Supplementary material for: The Impact of Instant Coffee and Decaffeinated Coffee on the Gut Microbiota and Depression-Like Behaviors of Sleep-Deprived Rats
Source: Front Microbiol. 2022 Feb 25;13:778512. doi: 10.3389/fmicb.2022.778512 (PMC8914519; doi:10.3389/fmicb.2022.778512)
Supplement: Supplementary file 1 [file Data_Sheet_1.DOCX]

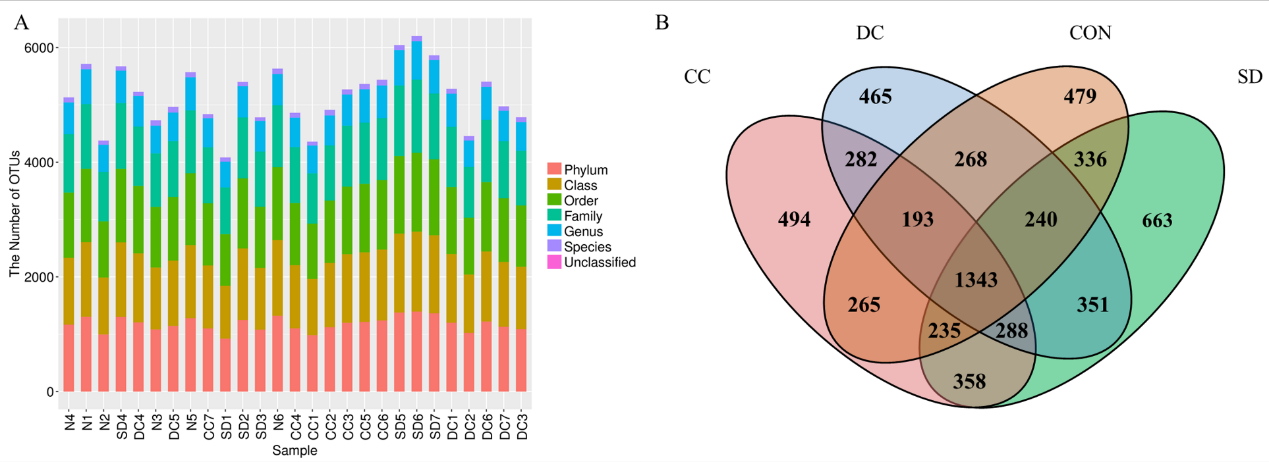


**Fig. S1 The number of OTUs among 4 groups.** The number of OTUS in different taxonomy among 4 groups (A). the venn diagrams among 4 groups (B). CON: control group; SD: PSD model group; CC: conventional coffee group; DC: and decaffeinated coffee group.
